# Differential diagnosis of acute psychosis after cocaine consumption: a case report

**DOI:** 10.1192/j.eurpsy.2024.1528

**Published:** 2024-08-27

**Authors:** J. Gimillo Bonaque, E. Arroyo Sánchez, P. Setién Preciados, C. Díaz Mayoral

**Affiliations:** Psychiatry, Hospital Príncipe de Asturias, Alcalá de Henares, Spain

## Abstract

**Introduction:**

Psychosis is a common clinical presentation of mental disorder in many psychiatric patients, however, an etiological diagnosis is important when it occurs for the first time in a patient. Regarding a case seen in the Emergency Department recently, with major depression and acute cocaine use, a differential diagnosis was made after adequate organic screening. When presenting delusion of infestation after the consumption of the substance, the main hypothesis was what we call Ekbom syndrome. However, among other possibilities we consider a toxic psychosis or a major depression with psychotic symptoms.

**Objectives:**

Review the different causes of acute psychosis and the importance of a good clinical history to achieve a specific diagnosis. Perform a differential diagnosis between the main causes of psychosis in a patient with depression who has recently consumed cocaine.

**Methods:**

Presentation of the case and review of the available literature on the risk of developing psychosis after cocaine use and depression concomitantly.

**Results:**

There is a low number of reported cases of delusional infestation after acute cocaine use, being more likely toxic psychosis or major depression with psychotic symptoms. A good anamnesis, with systematic questions about toxic habits, can lead us to a more accurate main hypothesis.

**Conclusions:**

We mark the importance of a systematic anamnesis to achieve a better diagnosis, as well as a correct study by the clinician of the specific syndromes described in phenomenology such as Ekbom syndrome, to make a correct association of ideas in the differential diagnosis.

**Disclosure of Interest:**

None Declared

